# CellML metadata standards, associated tools and repositories

**DOI:** 10.1098/rsta.2008.0310

**Published:** 2009-05-28

**Authors:** Daniel A. Beard, Randall Britten, Mike T. Cooling, Alan Garny, Matt D.B. Halstead, Peter J. Hunter, James Lawson, Catherine M. Lloyd, Justin Marsh, Andrew Miller, David P. Nickerson, Poul M.F. Nielsen, Taishin Nomura, Shankar Subramanium, Sarala M. Wimalaratne, Tommy Yu

**Affiliations:** 1 Auckland Bioengineering Institute, University of AucklandAuckland 1142, New Zealand; 2 Department of Physiology, Anatomy and Genetics, University of OxfordOxford OX1 2JD, UK; 3 Department of Physiology, Medical College of WisconsinMilwaukee, WI 53226, USA; 4 Division of Bioengineering, National University of SingaporeSingapore 117574, Republic of Singapore; 5 Department of Bioengineering, University of California, San DiegoLa Jolla, CA 92093, USA; 6 Department of Mechanical Science and Bioengineering, Osaka UniversitySuita, Osaka 565-0871, Japan

**Keywords:** CellML, markup languages, metadata, modelling

## Abstract

The development of standards for encoding mathematical models is an important component of model building and model sharing among scientists interested in understanding multi-scale physiological processes. CellML provides such a standard, particularly for models based on biophysical mechanisms, and a substantial number of models are now available in the CellML Model Repository. However, there is an urgent need to extend the current CellML metadata standard to provide biological and biophysical annotation of the models in order to facilitate model sharing, automated model reduction and connection to biological databases. This paper gives a broad overview of a number of new developments on CellML metadata and provides links to further methodological details available from the CellML website.

## 1. Introduction

CellML (www.cellml.org; Lloyd *et al*. 2004) is an extensible markup language (XML, www.w3.org/XML) being developed by the International Union of Physiological Sciences (IUPS, www.iups.org) Physiome and European Virtual Physiological Human (VPH) projects to encode mathematical models of biological processes that are based on systems of ordinary differential equations (ODEs) and algebraic equations—so-called ‘differential algebraic equations’ (DAEs). This has applications to all models of cellular processes where spatial gradients are ignored (spatial information is handled by a complementary standard called field modelling language (FieldML, www.fieldml.org), which is discussed by Christie *et al*. (2009)) and also to systems physiology models where a ‘lumped parameter’ representation is used. The language is designed to support the definition and sharing of models of biological processes by including information about: *model structure* (how the parts of a model are organizationally related to one another); *mathematics* (equations describing the underlying biological processes); and *metadata* (additional information about the model; see §2 below). CellML is built on existing standards such as content Mathematical Markup Language (MathML) for encoding the mathematics (www.w3c.org/Math) and the Dublin Core for bibliographic information (www.dublincore.org). The current release of the CellML standard is available at www.cellml.org/specifications. Note that CellML, with its focus on biophysical processes, is complementary to another XML language called Systems Biology Markup Language (SBML, www.sbml.org), which principally represents biochemical reaction networks and is widely used in the systems biology community.

**Figure 1:**
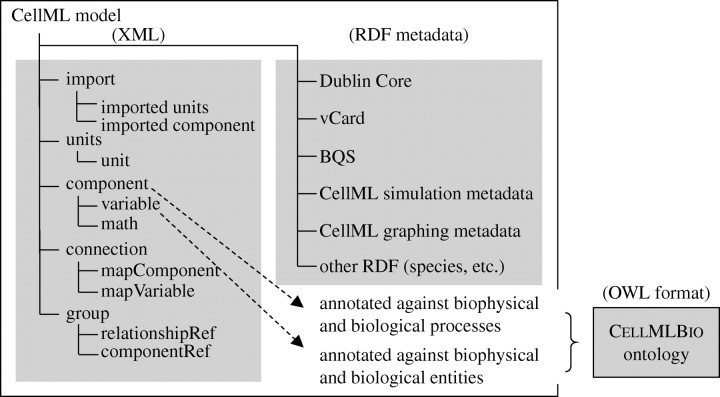
Entities in a CellML model. The CellML model file on the left contains the base model with its imports, units, components, connections and groups described in XML format, and the metadata in RDF format. The annotation of CellML variables with biological and biophysical meaning is handled via cmeta:id links to terms stored in RDF format in a separate OWL file (see §3).

CellML has a simple structure (figure 1) based on connected components. These components are abstract concepts providing well-defined interfaces to other components, and encapsulate concepts by hiding details from other components. Connections provide the means for sharing information by associating variables visible in the interface of one component with those in the interface of another component. Consistency is enforced by requiring that all variables be assigned appropriate physical units, the dimensions of which must match when variables are connected. Public and private interfaces enable encapsulation hierarchies, providing further mechanisms for information hiding and abstraction. Model reuse is facilitated by the import element, enabling new models to be constructed by combining existing models into model hierarchies. The CellML 1.1 standard is available at www.cellml.org/specifications/cellml_1.1.

The CellML Model Repository currently contains over 370 models from peer-reviewed publications of biological processes ranging from gene regulation, ion channel electrophysiology, signal transduction and metabolic pathways to bioengineering constitutive laws and larger scale systems physiology processes (www.cellml.org/models). Approximately half of these models have been curated to a level where they are internally consistent in their units and have all the necessary parameter values and initial conditions to numerically integrate successfully and give outputs that match those in the source publication (Lloyd *et al*. 2008). Freely available open-source CellML authoring and simulation software can be obtained from www.cellml.org/tools.

The CellML project is an international open-source effort involving input from many people. Every attempt is made to be as open and inclusive as possible in the decision-making processes.

Most of the current project activities are described as items in the Physiome Tracker (https://tracker.physiomeproject.org). Within the CellML project category, the tracker is organized according to subject area (i.e. the CellML specifications, CellML Model Repository, model repository software, etc.), and issues are able to be searched, sorted and organized according to attributes and keywords. Tracker items are usually open for feedback for a limited period of time. After the closing date, a panel of CellML members considers the tracker contributions and attempts to reach a consensus. If consensus is reached, one of the panel members will add a comment to the tracker item, describing the consensus and marking it as resolved. If the panel cannot reach consensus, the CellML project leader will either cast a deciding vote, or reframe and reopen the issue as she/he sees fit.

The CellML group at the Auckland Bioengineering Institute meets regularly (typically weekly). Although this group represents only a portion of the CellML community, it is engaged in most of the core projects, such as the development of the CellML specification, CellML-related software, the www.cellml.org website and curation of the CellML Model Repository. The recorded minutes for these meetings provide an indication of what issues related to CellML are currently being discussed.

The CellML email discussion lists provide one of the principal media for community members to discuss projects currently being worked on. Most community-related exchanges occur on the cellml-discussion mailing list (www.cellml.org/mailman/listinfo/cellml-discussion), a mailing list targeted at those interested in contributing to or providing feedback on the development of the CellML language.

The next major goal for CellML, to facilitate multi-scale modelling in the Physiome/VPH project (Hunter & Nielsen 2005), is the further development of metadata structure using the semantic web standards based on the Web Ontology Language (OWL, www.w3.org/TR/owl-features) and the Resource Description Framework (RDF, www.w3.org/RDF). The aim of the present paper is to outline the various developments that are underway for specifying CellML metadata. We discuss the process of annotating the model components with both biological and biophysical meanings such that: (i) new models can be constructed by importing older models or importing components from a library of submodels, (ii) biophysical constraints can be automatically checked (e.g. is the model consistent with the laws of thermodynamics?), (iii) visual representations of the models, such as pathway diagrams, can be generated automatically, (iv) model reduction algorithms can be applied to generate less complex models appropriate for use under particular circumstances, and (v) CellML models can be created directly from databases of, for example, molecular binding parameters in metabolic and signal transduction networks, including their dependencies on ion concentrations, pH, temperature, etc. We also discuss the development of a simulation metadata standard and a graphing metadata standard. Tables 1 and 2 provide an overview of the current status of standards and software mentioned.

**Table 1:** Standards: current status.

specification name	status	version
CellML specification	released	1.1
CellML metadata specification	public draft	draft
CellML graphing metadata specification	public draft	draft
CellML simulation metadata specification	public draft	draft
MathML	W3C recommendation	2.0
RDF	W3C recommendation	22 Feb 1999
Dublin Core	recommendation	1.1
SBML	released	level 2 version 4
OWL	W3C recommendation	10 Feb 2004
MIRIAM	proposal	unspecified
vCard	W3C submission	22 Feb 2001
BQS	finalized	version 2
MIASE	under development	pre-alpha
SED-ML	object model published	unspecified
FieldML	under development	n.a.

**Table 2:** Software: current status.

software	status	version
PCEnv	released	0.5
CellML API	released	1.5
COR	released	0.9
*insilico*IDE	released	0.2.5
JSim	released	1.6.84
PMR1	live	unspecified
PMR2	under construction	n.a.

We begin by discussing the current CellML metadata specification, the new annotation and visualization metadata requirements and the proposed simulation and graphing metadata standards. We then discuss two case studies for the use of CellML metadata, one on building signal pathway modules and the other on the integration of a biochemical database into the CellML framework. We end with a discussion of the new CellML 1.1 Model Repository and current CellML simulation tools. A further example illustrating the application of these technologies as a model publication aid is provided in Nickerson & Buist (2009).

## 2. The CellML metadata specification

CellML metadata serves two primary purposes: to describe what a model is and where it came from, and to annotate elements within the CellML document with relevant information. Metadata is also essential for distributing CellML models via any web-based repository. For a CellML document to be useful to the scientific community, it needs to meet certain requirements in terms of metadata. The minimal information requested in the annotation of biochemical model (MIRIAM, www.ebi.ac.uk/miriam) standard (Le Novère *et al*. 2005) defines minimum requirements for metadata annotation of biological models and is relevant to CellML models.

The current draft CellML metadata specification leverages several pre-existing metadata standards and creates some new CellML-specific definitions to provide a framework for defining metadata within a CellML document. These pre-existing standards include Dublin Core, vCard (www.imc.org/pdi/) and bibliographic query service (BQS, www.omg.org/cgi-bin/doc?dtc/2001-12-03).

Dublin Core is a set of metadata properties that were identified as common across a wide range of applications within library science and knowledge management, such as ‘creator’, ‘publisher’, ‘date’, ‘subject’, etc. vCard is an RDF definition of metadata about people and is used to annotate a CellML document with comprehensive information about the people who have been involved in all aspects of the model development. BQS provides a framework for defining bibliographic metadata. At the time the original CellML metadata specification was written, BQS was just a draft standard and no other standards existed for defining bibliographic metadata. BQS has since been superseded by frameworks such as the MIRIAM standard (Le Novère *et al*. 2005).

Modification history metadata can describe who made what changes to any resource contained in a CellML model and at what time, which is particularly important in determining the provenance of a model (see Nickerson & Buist (2009) for further discussion on the application of modification metadata). Currently, the addition of modification metadata is an optional step during the process of uploading a model to the repository, and the nature of this information is free form. Specific requirements for the modification metadata will be addressed in the near future by the new CellML 1.1 repository software (see §8 below).

In addition to modification history, CellML metadata can also be used to define alternative names for CellML elements, add information pertaining to the species and/or sex of the organism the model specifically describes, and can also be used for free-form comments, annotations and descriptions of elements.

The most essential metadata defines where the model came from: if it is a description of a model from the literature, what is the citation? Who encoded the CellML, at what time? This information can be described using vCard and Dublin Core qualifiers. This is the core compulsory set of metadata, which is required to be associated with a CellML model for it to be entered into the CellML Model Repository. This metadata allows the models to be associated with the citation from which they are derived and provides information on provenance and must be added when a model is uploaded to the repository. A Uniform Resource Identifier (URI, www.w3.org/TR/uri-clarification) is then derived from the author names, the publication date of the citation and the version number of the model. This URI is then associated with the model and converted to a Uniform Resource Locator (URL) where the model is stored.

The CellML metadata specification allows detailed revision histories to be associated with a model. As a model is curated, incremental changes to the model code are made. These changes may be trivial, such as correcting errors made during the translation of the model to CellML, or they may be more substantial, such as incorporating revisions to the model. For a model to be reused, it is essential that these changes are listed and fully documented so that a prospective user knows what has been changed and why, by whom and when; this information is especially salient when non-trivial changes to a model have been made. As model hierarchies defined using CellML 1.1 can be defined across multiple physical locations and components can be imported and reused from any of those locations, it is important to ensure that the modification annotations are associated with the appropriate resources in the model. It makes sense, for example, to have the history of parameter value changes associated with the corresponding variables in the submodel of the hierarchy in which the value is defined.

Other metadata that are relevant to curation of models in the CellML Model Repository include a brief description of the model, a comment on the curation status of the model and keywords about what the model describes. In the model publication 2.0 framework described in Nickerson & Buist (2009), a significantly more detailed description of the complete model is proposed, specified using not only the CellML metadata requirements described above for the CellML Model Repository but also the other metadata described in this paper including the CellML Simulation Metadata (CSM) standard, the CellML Graphing Metadata (CGM, www.cellml.org/specifications/metadata/graphs) standard and new proposals for model biological and biophysical annotation.

### (a) Support of the MIRIAM standard in the CellML metadata specification and curation practices

The MIRIAM standard (Le Novère *et al*. 2005) is a community-agreed framework that defines the minimum requested information for annotation of biological models. The essential tenets of this framework are threefold: reference correspondence; attribution annotation; and external resource annotation.

The ‘reference correspondence’ requirements dictate that the model be encoded in a ‘public, standardized, machine-readable format’ and that the model be fully constrained and solvable to reproduce the results published in the reference description of the model. The validity of these assertions with respect to a particular model cannot currently be represented using the CellML metadata specification, but an effort is underway to use ‘curation flag’ qualifiers to achieve this. Assigning curation flags to a model would be an improvement on the current curation system, which relies on general text comments and assigning the model a number of ‘stars’, which can lead to uncertainty. A more descriptive set of ‘flags’ may be more informative, for example, ‘units/dimensions consistent’, ‘gives same results as referenced publication’, ‘meets the MIRIAM standard requirements’, etc.

To satisfy ‘attribution annotation’ under MIRIAM, the full provenance of both the original model and the CellML document that describes the model must be documented. This includes the citation of the reference description of the model, the name and contact information of the model authors and curators and the date the model was created and last modified. A version history is not specifically required by MIRIAM; however, the CellML project considers this information essential. This attribution annotation information can be specified using Dublin Core, vCard and BQS.

‘External resource annotation’ mandates that every constituent of a model be unambiguously annotated to a piece of knowledge, such as a database entry or an ontological term. The MIRIAM resources initiative (Laibe & Le Novère 2007) provides a framework for this in the form of MIRIAM URIs. These provide ‘a way to uniquely describe entities with a perennial, stable identifier’, and to ‘link an entity to one or more online resources, where extra knowledge about it is available’. This information can be represented from within the CellML metadata specification using Dublin Core, although work is required to develop a best practice system.

MIRIAM URIs can also refer to citations, such as PubMed identifiers. The CellML project is currently considering the replacement of the BQS with MIRIAM URIs, as they are simpler and represent a much more widely accepted standard. By subscribing to such standards in CellML metadata, the potential for collaborative curation of biological models across the range of formats is greatly increased.

## 3. Annotation and visualization of CellML models

CellML models are usually structured in order to accurately represent the biophysical information associated with biological processes. In general, this biophysical information is more detailed than is necessary for representing biological information at a more abstract user-dependent level. Furthermore, there is generally not a one-to-one mapping between biophysically and biologically relevant entities. It is thus difficult to provide an automatic translation between the biophysical and biological representations of biological processes. There are ways, however, to facilitate this translation by linking entities in CellML models to biophysical, biological and other ontologies. A software framework for annotating and visualizing CellML models is being developed, which involves the following:

- An ontology for storing CellML models in OWL format (CellML/OWL) and a set of rules for binding these CellML/OWL instances to CellML models using the CellML metadata specification. The CellML/OWL model captures the CellML structure in OWL. The use of OWL also allows the CellML/OWL model to be easily integrated with external ontologies to provide additional information.
- An ontology that represents biophysical and biological concepts that are covered in CellML models (ontology for biophysical and biological concepts covered in CellML models, CellMLBio), together with a set of rules for binding these concepts to CellML/OWL models. The CellMLBio model can be linked to external vocabularies such as the Systems Biology Ontology (SBO, www.ebi.ac.uk/sbo; Le Novère *et al*. 2006), the Gene Ontology (www.geneontology.org) and BioPAX (www.biopax.org) to take advantage of existing biological model descriptions. The biophysical and biological annotations can provide sufficient information to enable graph reduction rules to be applied to the CellMLBio model to generate a representation of the underlying biological model.
- A specification for building visual glyphs that support a visual language which can be used to represent all biophysical and biological processes covered in the CellMLBio ontology. The current workflow uses a novel visual language that is being developed specifically to represent the concepts modelled in CellML.
- An ontology for storing the references to the visual language (Visual Template Ontology, VTO) together with the rules for binding the visual language to concepts within the CellMLBio ontology. This method also allows modellers to visualize biochemical pathways represented in CellML models using other notations such as the Systems Biology Graphical Notation (SBGN, www.icsb-2007.org/proceedings/abstracts/G19.pdf) by extending the VTO.
- A visual editing tool that combines the visual language and ontologies in order to visualize CellML models.

**Figure 2:**
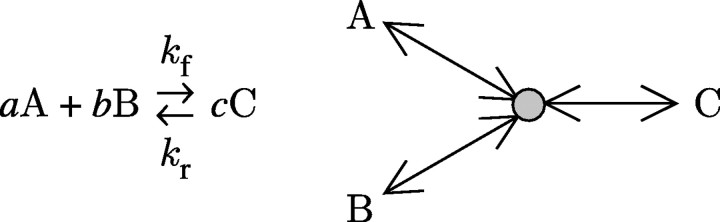
A simple reaction.

To illustrate the distinction between biological and biophysical annotations and how these are tied together with the CellML metadata, consider the simple reaction shown in figure 2 in which *a* molecules of a protein A combine with *b* molecules of a protein B to produce *c* molecules of a protein C with a forward reaction rate of *k*_f_ and a reverse reaction rate of *k*_r_.

The workflow for annotating the CellML model describing this simple reaction and generating diagrams is as follows.

- Construct a CellML model clearly separating the biophysical concepts. Three CellML components are created to contain the mathematical equations representing the rate of change of concentrations (i.e. a flux *J* is the rate of change of a concentration [.]): component A, d[A]/d*t*=*J*_A_; component B, d[B]/d*t*=*J*_B_; and component C, d[C]/d*t*=*J*_C_. Note that three new variables, *J*_A_, *J*_B_ and *J*_C_, have been introduced. To represent the flux balance, another CellML component is created: component D, *J*=*k*_f_[A]^*a*^.[B]^*b*^−*k*_r_[C]^*c*^; *J*_A_=−*aJ*; *J*_B_=−*bJ*; and *J*_C_=*cJ*. CellML ‘connections’ are made between components A, B and C and component D.
- Transform the CellML model into OWL format using the CellML/OWL ontology.
- Generate a CellMLBio model that identifies components and connections as physical processes, and variables as physical entities.
- Using the CellMLBio ontology, at the physical level type variables as physical entities (concentrations or fluxes) and the components as physical processes (‘pooling’ and flux balance with mass action kinetics), and at the biological level type variables as biological entities (proteins) and the components as biological processes (biochemical reaction). For example, the three variables A, B and C are annotated at the biological level to be proteins A, B and C and at the physical level to be the concentrations [A], [B] and [C] with units of mM (in an ontology sense these are ‘instances’ of the physical concept ‘concentration’). Map these CellMLBio model instances to VTO instances to provide visual information. This annotated CellMLBio model can be used to generate a physical view.
- Create a biological model by applying ‘reducing rules’ that map the biophysical entities such as [A] and *J*_A_ to the same biological entity, in this case the protein A. This provides a mechanism for displaying biological pathway diagrams from the biophysically related mathematical models. Importantly, the biological entity retains pointers to all the biophysical instances that have been collapsed into it.

**Figure 3:**
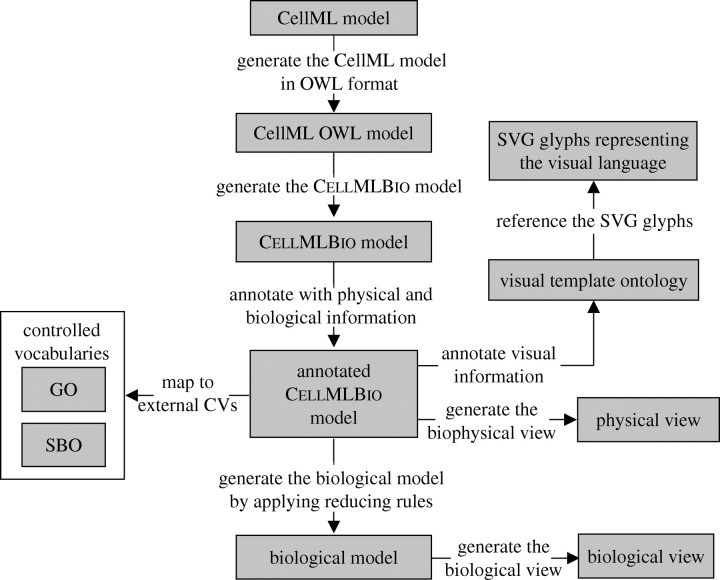
Workflow representing the process of annotating and visualizing CellML models. Note that SVG is the XML-based ‘Scalable Vector Graphics’ standard used in CellML simulation tools such as PCEnv (see §9).

**Figure 4:**
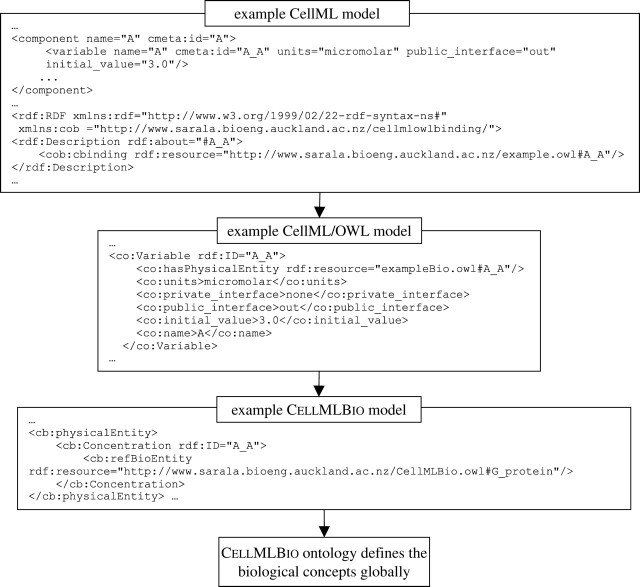
Using the metadata specification to annotate CellML elements to biological concepts.

Figure 3 illustrates the workflow associated with these steps, and figure 4 illustrates the way in which the CellML metadata specification is used to annotate the CellML elements to biological concepts. RDF code maps variable A_A defined in the CellML model to the A_A instance defined in the CellML/OWL model. CellML/OWL model instances are mapped to the CellMLBio model instances via OWL properties defined in CellML/OWL.

## 4. Simulation metadata

The CSM specification, which currently exists as a public draft at www.cellml.org/specifications/metadata/simulations, provides metadata about how simulations can be carried out from CellML models. The focus is on simulations of how DAE model variables evolve with respect to a single variable (often time).

By providing CSM, which in turn references a CellML model, it becomes possible to describe precisely how a particular numerical output, which may be discussed in a publication for example, can be generated. It also allows model users to exchange enough information to repeat a simulation, even when they are using different modelling tools and environments.

Efforts are currently underway to develop a specification for ‘minimum information about a simulation experiment’ (MIASE, www.ebi.ac.uk/compneur-srv/miase). Proposed MIASE guidelines state that four types of information should be provided: information about the models simulated; the simulation methods used; the task performed; and the outputs produced. CSM references a CellML model by its URL. It also allows for the specified simulation method to be described, by reference to a controlled vocabulary of algorithms, and provides information on the parameters of each simulation algorithm, such as error tolerances. In addition, information about the simulation task, such as the range (e.g. of times) over which the simulation is to be carried out, can be specified. Information about graphical outputs produced is not specified in CSM, but is instead described using CGM (discussed in more detail below). These two specifications can therefore be used together to achieve a MIASE-compliant description of a simulation experiment.

Another specification currently in draft form Simulation Experiment Description Markup Language (SED-ML, www.ebi.ac.uk/compneur-srv/sed-ml), which has been developed by the SBML community to tackle a problem similar to that of CSM. Unlike CSM, SED-ML has been designed to allow references to models in different formats. At its present state of development, however, it cannot be used to support references to CellML models, as it only supports modelling languages where time is an intrinsic concept of the language. In CellML, time is not special, but is instead often explicitly identified as a variable.

## 5. Graphing metadata

The proposed CGM specification (www.cellml.org/specifications/metadata/graphs) provides metadata about how to extract specific summaries of simulation results. These simulation observations are obtained by performing the numerical simulations required by the graph definition and are generally specified using CSM. Simulation observations may include simulation data from many different mathematical models using many different parametrizations of each of the models; or they may simply illustrate the dynamical behaviour of a single variable.

Initially, the primary objective for graphing metadata was to be able to completely describe typical journal article simulation outcome figures in a machine interpretable and software agnostic format. Such a specification agrees with the MIASE guidelines for information on the outputs produced. This initial objective has since evolved into a more generic framework for the definition of any pertinent simulation observation as part of a comprehensive description of a model's development, validation and application (Nickerson & Buist 2008; Nickerson *et al*. 2008).

In addition to specifying the data to be extracted from simulation outputs, graphing metadata also provides the model author with the ability to explicitly connect simulation observations to external data. Such external data could be experimental observations used in model development or validation/curation. The external data could alternatively be curated simulation observations, used to validate and curate independent software tools as an aid to quantitatively evaluate the abilities of each tool to correctly interpret and simulate the described models. This feature provides a powerful tool for the automation of model validation and curation workflows, with software able to automatically compare simulation outputs directly with either simulated or experimental data. In the field of bioinformatics, much has been achieved in the area of standard formats for the archiving and retrieval of experimental data, most notably the vast amounts of protein expression data obtained from high throughput assays. Similar standards are lacking in areas closer to those usually encompassed by CellML models and will need to be established in order to make full use of the power of CGM.

While generally used in conjunction with mathematical models encoded in CellML, there is nothing specific to CellML in the definition of the CGM specification. All references to model variables and numerical simulations are explicitly defined using URIs, which allows software to interpret the referenced resource as best as it can. This is seen as an advantage of CGM, as it can be used with other model encoding formats such as SBML (Hucka *et al*. 2003).

## 6. Case study 1: building signalling pathway modules

Model metadata will greatly facilitate the construction of models of signal transduction pathways, particularly when developing a model of the system of interest by reusing and aggregating components (submodels) previously constructed and validated by the scientific community. Metadata standards should be developed for all stages in the model construction workflow, aiding the selection of components for inclusion, the linking of submodels together and the parametrization of the final model for the new questions of interest and the accompanying cellular state.

### (a) Metadata aids component selection

When selecting model components for inclusion in a model, it is crucial to ensure that the components used are from appropriate cell types as much as possible. Biological process differences between species, life cycle and cell lines (Lukas 2004), and even gender (Trepanier-Boulay *et al*. 2001) of host organisms, can be significant to the questions of interest, and therefore the model construction process. Each of these criteria represents a dimension that should be annotated with metadata. A model for a co-transporter in male, neonatal, rat cardiomyocytes should be identified as such, and therefore it is distinct from a model for the ‘same’ co-transporter in female adult fruitfly gut stem cells. Identification annotations should be as specific as possible, but more abstract identification can be inferred from the ontological definition of the identification terms (a male neonatal rat is a mammal, for example). Where possible, models for the exact desired cellular situation are used, but it is common practice still to use measurements and also model components from related cell types that are assumed to be similar. The purpose of cell-type metadata should therefore not be to enforce complete compliance between the biological system and the model components, but certainly to make it clear what kind of components are being used and therefore the structural assumptions in the aggregated model.

**Figure 5:**
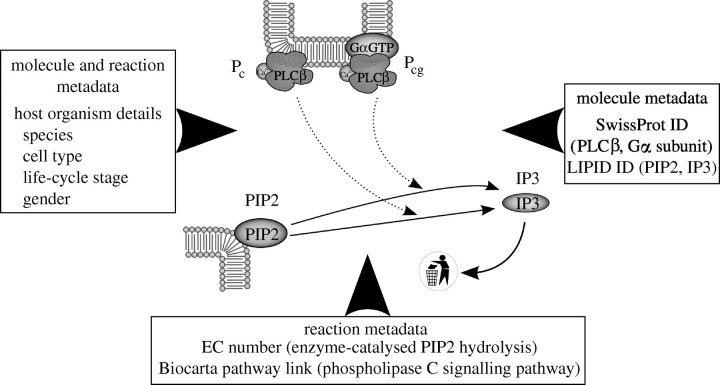
Example metadata for a simple model of phosphatidyl inositol 4,5-bisphosphate (PIP2) hydrolysis derived from Cooling *et al*. (2007). P_c_ is phospholipase C with calcium attached and P_cg_ is phospholipase C with calcium and also a G-protein alpha subunit activated by guanosine triphosphate (GTP). IP3 is inositol 1,4,5-trisphosphate.

In the construction of models from modular CellML models, the components will represent specific molecular species, fluxes and reactions (Cooling *et al*. 2008). Using metadata, models of reactions could be linked to standard repositories of reactions, e.g. by enzyme nomenclature number for enzyme-catalysed reactions (www.chem.qmul.ac.uk/iubmb/enzyme). Molecular species, such as diffusible proteins, could be linked to SwissProt accession numbers (www.ebi.ac.uk/swissprot) or Protein Data Bank (PDB) identifiers (www.wwpdb.org). These could be used as search keys for pre-built components of molecules of interest. To aid understanding of the context of biological entities and reactions, links to publicly available pathway diagrams such as those provided by KEGG pathway (www.genome.jp/kegg/pathway.html) or BioCarta (www.biocarta.com) would also aid model component selection by helping the model builder decide what other biological processes and entities may be apposite to the aggregated model. Example metadata for a step in the phospholipase C signalling pathway is shown in figure 5.

Metadata on the structural and behavioural aspects of the model will also aid component selection. Structural information embodied in curation metadata, such as the internal consistency of units and consistency with source publication (if any) are helpful for assessing the readiness of a model component and the assumptions under which the model will perform as described. Since source publications very often do not describe their models accurately (Le Novère *et al*. 2005), descriptions of exactly what the model does are crucial to the component selection process in building new models. CSM and CGM will be invaluable in making these descriptions explicit, as demonstrated in Nickerson & Buist (2009).

### (b) Metadata aids component linking

CellML's insistence on units for quantities greatly reduces the likelihood of incompatible variables being linked together (such as a flux in one component being linked to a molecular species in another). Nevertheless, without metadata, it would be possible to erroneously link different fluxes together, or link the concentration of an enzyme to the concentration of a second messenger ion. Ensuring that components are appropriately *semantically* linked probably requires human intelligence, but the process could be greatly assisted by metadata linking to appropriate ontologies that clearly define what variables represent. Such linkages would aid software tools in notifying model builders if, for example, an enzyme-catalysed reaction had its ‘enzyme’ variable linked accidentally to a diffusible messenger ion instead. Such annotation also aids in the integration of CellML models into other Physiome standards, such as automating the integration of CellML models of cellular electrophysiology with models of electrical activation in the whole heart using FieldML (Christie *et al*. 2009).

### (c) Metadata is crucial for model parametrization

When forming models from a set of model components, care must be taken that the parameters of the model are still appropriate. Additionally, applying the model components to a new situation probably requires some parameters to change, and these changes should not inadvertently violate assumptions implicit in the components' formulations. Metadata can be used to describe these assumptions, which may be quite simple, as in these examples,

- variable ‘A’ should be in the range *x*–*y* and
- variable ‘A’ should be in the range *x*–*y* if parameter ‘B’ is between *s* and *t*.

These ranges may also be dependent on cell-type information, such as those described above, which yield physiological ranges for parameters in different cells. Variable value assumptions may be more complex, such as

- if variable ‘A’ is changed, then variable ‘B’ should be adjusted so that at steady state the model reads ‘*x*’ for parameter ‘C’.

An example of this last point in the context of the model depicted in figure 5 is that there the steady-state concentration of inositol 1,4,5-triphosphate (IP3) should always be 15 nM. If the total calcium or phospholipase C levels are altered (such as by adding a phospholipase C activation cycle model from Cooling *et al*. (2008)), it will impact on the steady-state concentration of IP3. Under the assumptions of the model construction (Cooling *et al*. 2007), the forward rate constant for hydrolysis of phosphatidyl inositol 4,5-biphosphate (PIP2) by P_c_ must then be adjusted until the steady-state concentration of IP3 is 15 nM.

As with component linking, metadata facilitates tool-based identification and notification when parametrization leads to violation of these kinds of model assumptions.

## 7. Case study 2: integrating physical chemical data

Biological markup languages and associated tools are making it feasible to store, reproduce, merge and analyse computational models of increasing complexity and realism which rely on increasingly diverse sets of data for model construction, parametrization and validation. Important examples include realistic simulations of cellular metabolism, which are built on thermodynamic models of biochemical reactions and kinetic models of the enzymes catalysing those reactions. The thermodynamic models, used to estimate reaction thermodynamic properties at physiological temperature, ionic strength and ionic content, are constructed from raw data on ion dissociation and chemical equilibria under a variety of *in vitro* conditions. Similarly, the catalysis models are constructed from kinetic data obtained under a variety of (non-physiological) *in vitro* conditions. Reliable and realistic simulations of biochemical systems that are consistent with physical chemical theory, and these diverse data on kinetics and thermodynamics, can be constructed in a unifying theoretical/computational framework (Vinnakota *et al*. 2009). Software tools for robust modular organization of data and model of this sort are currently under development.

### (a) Techniques for simulation of biochemical systems

**Figure 6:**
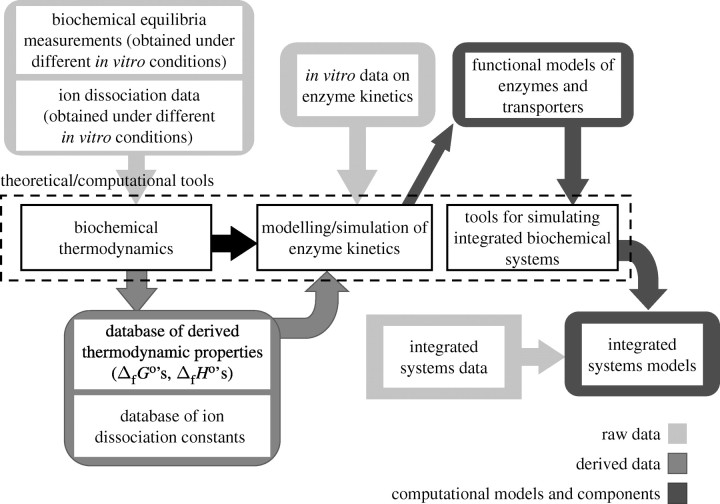
Relationships between the raw dataset, derived data and biological models in biochemical system modelling.

Figure 6 illustrates the critical dependencies between raw datasets, theoretical tools and technology, and derived data and computational models in biochemical system modelling. Raw datasets include low-level chemical data (on ion dissociation and reaction equilibria) and data from biological components (such as purified enzymes) and function systems (such as cells, tissues or organs). A variety of theoretical and computational tools are applied to analyse these raw data to generate sets of derived data and models of biological systems.

As indicated by the arrows in figure 6, databases of estimated ion dissociation constants and free energies of formation (Δ_f_*G*°'s) and enthalpies of formation (Δ_f_*H*°'s) for biochemical species are constructed from raw data on biochemical reaction equilibria and association/dissociation data for relevant ionic species. Two efforts by the US National Institute for Standards and Technology (NIST, www.nist.gov) are resources for these data. First, the NIST database of thermodynamics of enzyme-catalysed reactions (NIST Standard Reference Database 74; http://xpdb.nist.gov/enzyme_thermodynamics) provides a collection of raw data on biochemical reaction equilibria (Goldberg *et al*. 2004). Second, the NIST database of critical stability constants (NIST Standard Reference Database 46, www.nist.gov/srd/nist46.htm) provides a curated collection of data on cation-biochemical species dissociation constants (Martell & Smith 2003). The theory of biochemical thermodynamics provides the tools for analysing and interpreting these data, accounting for pH, temperature, ionic strength and ion content. Of particular value here is the work of Alberty, who has not only made a number of important theoretical contributions, but also developed a database of derived thermodynamic data (Alberty 2003). Alberty's thermodynamic database and NIST Standard Reference Database 46, while incomplete, represent the richest available sources of fundamental thermodynamic constants for biochemical system models. They provide the basis for computing biochemical reaction equilibrium constants (and equilibrium-free energies) as functions of biochemical state and for computing the distributions of biochemical reactants into rapidly converting species (e.g. how adenosine triphosphate (ATP) is distributed among ATP^4−^, HATP^3−^, MgATP^2−^, etc. as a function of the solution conditions.) The basic theory of biochemical thermodynamics is described in Beard & Qian (2008*a*).

These databases are of fundamental value in analysing *in vitro* experiments on enzyme kinetics to develop simulations of how enzymes function *in vivo*. This is because *in vitro* kinetic experiments can rarely (if ever) be conducted under physiological cellular conditions. Yet, it is not feasible to obtain enough *in vivo* data to identify system models of the complexity necessary to simulate, for example, cellular metabolism. In practice, data from *in vitro* experiments are typically used to determine apparent mechanisms and associated parameter values for individual enzymes and transporters in a system. A handful of recent studies have analysed *in vitro* data on enzymes to develop models that account for distributions of biochemical species and the associated impact on kinetics (Beard *et al*. 2008; Qi *et al*. 2008), and more similar studies are underway.

Models of functional components (enzymes, transporters, etc.) that are constructed in this way can be seamlessly integrated into physico-chemically rigorous large-scale system models. The theory for integrating thermodynamically balanced models of biochemical system components that account for biochemical state and species distributions is described in recent publications (Beard & Qian 2008*b*; Vinnakota *et al*. 2009). Once again, the critical issue is that large-scale simulations account for biochemical species distributions and, consequently, dynamic buffering of metal and hydrogen ions.

Integrated models constructed under this framework typically require additional parameter estimation (mainly to estimate *in vivo* enzyme activities not available from the *in vitro* data) and model validation by comparing model predictions with data not used for parametrization. An example of a large-scale model developed under the framework of figure 6 is the cardiac energetics model of Wu *et al*. (2007, 2008). This model enjoys the significant advantage that not only does it match the metabolic data from quenched kinetics of purified mitochondria and *in vivo* ^31^phosphate magnetic resonance spectroscopy that were used to parametrize and validate it, but it is also consistent with the fundamental physical chemical theory of biochemical thermodynamics and associated data.

### (b) Tools for simulation of biochemical systems

Although the theories for physico-chemically rigorous simulations of biochemical systems are firmly established and straightforward, and a great deal of data are available for constructing such simulations, in the past these theories and data were almost universally ignored in biochemical system modelling. This is because building models at this level of detail requires an enormous amount of manual work in (i) assembling the disparate sorts of data, (ii) coding formulae and algorithms for biochemical thermodynamics and complex equations for dynamics buffering of metal and hydrogen ions in addition to the more standard chemical kinetic equations, and (iii) integrating all of these together in a self-consistent (bug-free) simulation package. Furthermore, with current tools, much of the coding needs to be redone to construct a new model, to integrate existing models together or even to update individual component of an existing model.

To make the technology and data for biochemical system modelling more widely available and more easily adopted, the framework illustrated in figure 6 is being integrated with the suite of CellML/Physiome tools. The components are as follows.

#### (i) Biological components databank

A biological components databank (www.biocoda.org) is being constructed to provide an online clearing house of mechanistic models of functional biological components to use as building blocks to construct computational models of biological systems. In contrast to other resources such as the Comprehensive Enzyme Information System (BRENDA, www.brenda-enzymes.info) and SABIO-Reaction Kinetics (SABIO-RK, http://sabio.villa-bosch.de) that compile reported estimates of kinetic constants obtained from the original sources, the approach here is to reanalyse reported data and determine mechanisms and associated parameter estimates for a number of enzymes and transporters. Constructing a truly useful resource along these lines will require widespread collaboration in the community.

#### (ii) Software for automated model construction

Software is being developed to automatically parse the data from the thermodynamic databases and the functional component (enzymes and transporters) model database into a CellML model. Because the ODEs used to describe these models can easily be constructed by intelligent algorithms, software to automatically generate models will save time and reduce the possibility of human errors in developing the equations and associated code.

Based on these tools, integration of data and biosystems modelling will be more seamless than is possible now. For example, with current tools, when some element of raw data (such as raw thermodynamic data) that appears early in the model development pipeline of figure 6 is added or updated, there is no mechanism for automatically passing this update downstream through the stages of model development. With all of these model development steps integrated into a single analysis platform, the updating and refining of biosystems models can be carried out automatically and self-consistently, providing a powerful platform for developing models of ever-increasing realism and complexity.

#### (iii) Generating CellML models from databases by means of populating model templates

Preserving metadata: when generating a CellML model from a database, metadata annotations could be included in the generated model, appropriately associated with the generated mathematical expressions.

The database could make use of a library of CellML components. The database would indicate the incorporation of a CellML model for each biochemical reaction. More than one model might be present in the library for a given reaction, and the database would indicate the preferred model. The preferred model would be imported. This allows alternatives to be used, i.e. these are ‘pluggable’ components.

Instead of using scripts to define how a model is assembled from the database, a template CellML model would be used. Template CellML models would also be present as components in a CellML library. The overall generated model could be specified by means of combining the appropriate templates, and populating template parameters appropriately. Some level of scripting could still be used, but the preference would be to use templates with metadata reasoners rather than custom scripting.

## 8. The CellML 1.1 Model Repository

The current CellML 1.0 Model Repository, based on models from peer-reviewed publications, now includes over 370 models of biological function ranging from gene regulation, cell signalling, membrane ion channel electrophysiology and metabolic networks to systems physiology models of blood pressure control and pharmacokinetic models (www.cellml.org/models). Over half the models in the repository have been curated to the point where they reproduce the results given in the source publication (many published models contain typographical errors or missing parameters and the CellML curators often work with the authors of the model to iron out these problems; Lloyd *et al*. (2008)). Open-source software is available (www.cellml.org/tools) including an application programming interface (API, www.cellml.org/tools/api) to read and write CellML models into software packages and simulation software that can be run either stand-alone or by clicking on a particular model in the repository.

The repository data are currently managed by a software system called the Physiome Model Repository (PMR, www.cellml.org/models). The PMR itself does not maintain version histories, but relies on the use of CellML metadata within each CellML file. This only offers tracking of document-level modification, i.e. one CellML model file at a time. PMR also provides a curation ranking system, where each model can be given zero to three stars based on some standardized criteria. The limitations of these two methods have prompted the design of a new system called PMR2 (www.cellml.org/tools/pmr2). An initial prototype of PMR2 has been built and tested in an informal trial, and the production version is under development.

PMR2 will make available an additional version history system for the repository models. It will allow version history information that pertains to groups of CellML files to be maintained, so that ‘snapshots’ of a group of files can be retrieved, i.e. retrieval of a group of model files as they appear at a specific point in time. This will be especially useful for CellML 1.1 models, since the use of imports requires a group of model files to be consistent with one another. The implementation of this feature in PMR2 will use a distributed version control system (DVCS; software traditionally used for software code version control), to track changes and to support enhanced collaboration between model authors. Information pertaining to each version in the history (modification time, author/user, parent version of model, etc.) forms part of the model's metadata.

Use of the original PMR has shown that using the preset numeric system to represent the quality of a model is too rigid, with no way to represent quality levels of separate quality criteria independently. PMR2 will introduce a curation flag system, where a tag with a value is associated with each model in the repository. The tags will be simple terms that represent the different aspects of a model (e.g. ‘model loads successfully into software X’), which are evaluated as part of the curation process, and the value is a Boolean score (i.e. yes or no) for the model with respect to that aspect. This will allow precise indication of the model's quality, specific searches based on these flags, and metrics of the overall quality of the repository, which can be tracked with time. These curation scores thus also form part of the metadata for a model.

## 9. CellML simulation software

Physiome CellML Environment (PCEnv), Cellular Open Resource (COR), *insilico*IDE (www.physiome.jp/downloads) and JSim (http://nsr.bioeng.washington.edu/jsim) are some of the available CellML editing and simulation environments (www.cellml.org/tools). They all offer a selection of core features: a selection of integrators and the ability to graph or save the output of a simulation.

PCEnv and COR also offer graphical rendering of equations, units and other parts of models. COR is designed to be a modal environment, with distinct editorial and computational modes. In the editorial mode, COR uses a human-readable syntax for editing of CellML. In the computational mode, the integrator and graphs can be configured, and simulations run. Further discussion of COR is available in Garny *et al*. (2009). PCEnv is non-modal. It allows for editing of CellML in several views: in a graphical tree that replicates the structure of a CellML file; as a list of variables with initial values; as a list of mathematical equations; and as raw XML. Some index 1 DAEs can also be solved by PCEnv v. 0.5. *insilico*IDE is an editing and simulation environment that can import and export CellML. It is a graphical editor; modules, edges and ports are the major parts of its visual representation of a model. Modules are represented by glyphs, ports by circles on the glyphs and edges by lines between either modules or ports. Modules are the aggregate components of a model, edges denote relationships, such as equality of variables or encapsulation, and ports expose parts of a module, such as variables. A summary of the capabilities of CellML tools is given in table 3.

**Table 3:** Matrix of features of note of CellML simulation software.

	COR	*insilico*IDE	JSim	PCEnv
model editing style	textual language	two-dimensional graphical editor	textual language	hierarchical trees, raw XML
CellML versions supported	1.0	1.0	1.0	1.0, 1.1
CellML file support	native	import, export	import	native
validation	syntactic, units	none	none	syntactic, units
procedural code export	yes; a variety of languages	yes; C++	no	yes; a variety of languages

**Figure 7:**
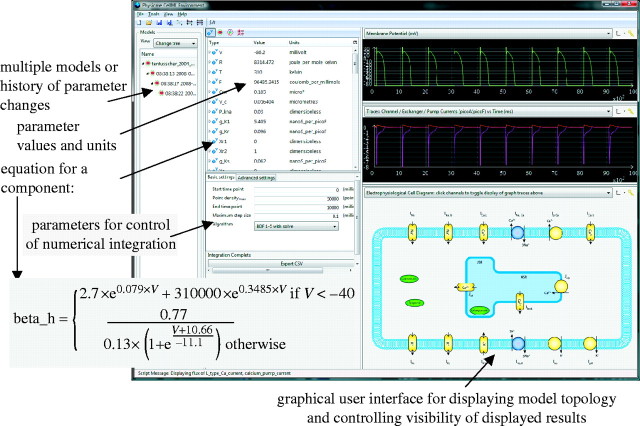
A screen shot of the freely available, open-source software PCEnv. This is one of a number of software programs that read and write CellML files and run simulations (www.cellml.org/tools). PCEnv now includes unit checking and can output code in a variety of computer languages (C, Fortran, Matlab, etc.). The particular model displayed here is the ten Tusscher cardiac electrophysiology model—see www.cellml.org/models/tentusscher_noble_noble_panfilov_2004_version05.

PCEnv can also be used for the display of models through the use of session files. PCEnv session files are a serialization of CGM together with PCEnv-specific information. The PCEnv-specific data allow for the association of external resources, such as Scalable Vector Graphics (SVG) diagrams, with a collection of CellML models and graphs when the session is displayed. This allows for the display of a visual representation of a model together with graphs generated from the model (figure 7).

The session files in the CellML Model Repository often associate SVG files with the models that they represent. SVG is a graphics format that is designed for interactivity; to this end, SVG has an event model, and SVG files can contain embedded JavaScript. PCEnv exposes a JavaScript API which can be accessed from within these SVG files; the common usage for this is setting a glyph to respond to being clicked on by toggling the displayed state of the relevant graph trace for the species it represents. This provides an association between a visually represented part of a model and the simulation results for that part of the model, making it easier for a viewer to navigate the model; they no longer need to know that the variable *x* corresponds to the flux of some particular species to retrieve the simulation results for that species, as the diagram can be used to toggle the display of the various simulation results.

## 10. Discussion

CellML is rapidly gaining acceptance as the standard for encoding biophysically based ODE or DAE models of biological function at the cell and organ system level. It may be used together with FieldML for anatomically based models where structure and spatial gradients are important. The comparable standard for biochemical networks is SBML, which has widespread acceptance in the systems biology community. To facilitate multi-scale modelling and to provide stronger links between the computational physiology community and the systems biology community, new metadata standards are proposed here that specify how model components can be annotated with biological and biophysical information. These annotation standards, together with simulation and graphing metadata standards and bibliographic information, will form the new CellML metadata specification. As much as possible, these metadata standards will be consistent with the ‘minimal information’ MIRIAM standards supported by both the CellML and SBML development groups.

The benefits of providing biological and biophysical annotation to CellML models, together with specifications for simulation and graphing, are many: it will enable the application of automated parameter estimation and parameter sensitivity analysis with CellML models and automate the generation of diagrams that illustrate the topology and biological meaning of CellML models. For some applications, such as metabolic pathway modelling, it will also enable CellML models to be generated automatically from databases of reaction kinetics, such that appropriate biophysical constraints (mass conservation, thermodynamic consistency, etc.) can be ensured as part of the model building process. Finally, it will greatly facilitate the building of databases containing parameter sets associated with different species or cell types, etc., for a particular CellML model.
